# Unsupervised End-to-End Deep Model for Newborn and Infant Activity Recognition

**DOI:** 10.3390/s20226467

**Published:** 2020-11-12

**Authors:** Kyungkoo Jun, Soonpil Choi

**Affiliations:** 1 Department of Embedded Systems Engineering, Incheon National University, Incheon 22012, Korea; 2 ChoisTechnology, Incheon 21984, Korea; sampo@choistec.com

**Keywords:** human activity recognition, deep learning, image processing, unsupervised, newborn

## Abstract

Human activity recognition (HAR) works have mostly focused on the activities of adults. However, HAR is typically beneficial to the safety and wellness of newborn or infants because they have difficulties in verbal communication. The activities of infants are different from those of adults in terms of its types and intensity. Hence, it is necessary to study the behavior of infants separately. We study newborn and infant activity recognition by analyzing accelerometer data from the sensors attached to body. We aim to classify four types of activities: sleeping, moving in agony, moving in normal condition, and movement by external force. For this work, we collected 11 h videos and corresponding sensor data from 10 infant subjects. For recognition, we propose an end-to-end deep model using autoencoder and k-means clustering, which is trained in an unsupervised way. From a set of performance tests, our model can achieve 0.96 in balanced accuracy and F-1 score of 0.95.

## 1. Introduction

Human activity recognition (HAR), which makes use of movement sensors such as accelerometer and gyroscope has been studied for a long time [1]. Recent breakthroughs of deep learning have contributed to significant success in HAR. Most research subjects of HAR have been adults. We believe that infants who have difficulties in verbal communication can benefit by applying HAR to monitoring their activities for their safety and wellness. 

For instance, the risk of sudden infant death can be reduced by monitoring a newborn’s sleeping posture using a 3-axis acceleration sensor [2]. A CO_2_ sensor is used to monitor the newborn’s breathing for abnormalities [3]. A triaxial accelerometer was used to detect abnormal wave motion of a bathtub and give a warning to guardians to prevent the infant drowning in the bathtub [4]. A sensory baby vest was proposed to measure the pulse oximetry and detect the motion and the prone position of infants with fully integrated sensors [5]. A gaze tracker [6] for behavioral analysis was proposed to monitor gaze and head movements in infants to evaluate head–eye coordination. A movement sensor was used to analyze the effect of the holding behavior on the relationship between newborn babies and parents [7]. In order to monitor the child’s play behavior, a smart jacket equipped with multiple movement sensors was also proposed [8]. In such monitoring, wearable sensor systems are preferred because they are unobtrusive and non-invasive. In addition, wearable systems utilize wireless communication systems for data collection, which is convenient in the healthcare of infants [9,10].

Our work focuses on newborn and infant activity recognition by analyzing accelerometer signals coming from a device attached to infants. For instance, movement sensors attached to infants enable sleep pattern analysis for health care and monitoring of external shock for safety. Since infant movement is considerably different from that of adults in terms of type and intensity, existing methods for adults have limitation on their application for infant cases. According to the classification guidance of WHO [11], newborn refers to babies who are within 28 days after birth, and infants are from 1 month old up to 12 months. We develop a method which can classifies the activity types from the sensor signals. We aim to tell whether subjects are asleep, how strong movements they show, and whether there are external forces that affect them.

**Figure 1:**
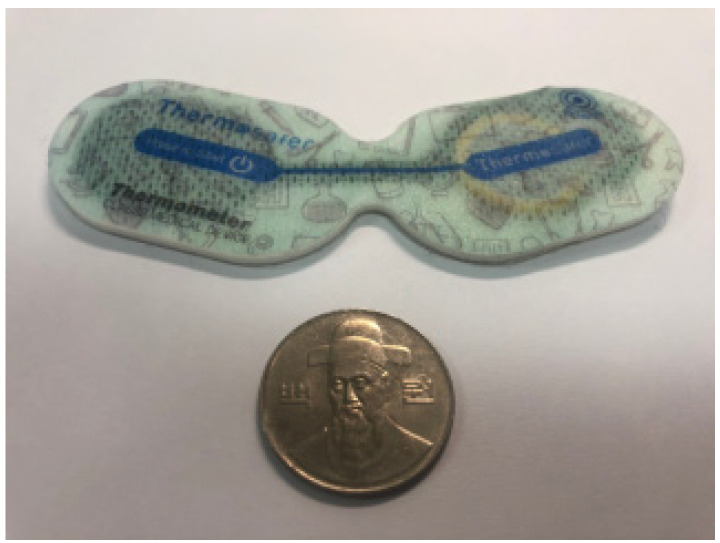
We use a wearable sensor which includes a thermometer and a 3-axis accelerometer along with Bluetooth transmission capability.

We use a commercial sensor device [12] shown in Figure 1. The device can be attached to clothes by using the adhesive back. It consists of a thermometer sensor and a 3-axis accelerometer along with a Bluetooth capability which transmits measured data. The accelerometer can measure movement at 40 Hz, while the thermometer operates at lower 0.25 Hz because of less frequent changes in body temperature. The device can operate continuously at least 24 h with an embedded battery.

For most HAR works which depend on sensor signals, deep learning methods are very popular because of its overpowering performance. As the complexity of activity classification increases, the tendency to depend on the deep learning also enlarges. For professional medical use, it is used to detect tremor caused by Parkinson’s disease [13] and classify complex behaviors that occur simultaneously [14]. Additionally, since wearable systems rely on the battery, tasks that affect battery life are classified using deep learning [15].

However, most deep learning methods accompany the burden of data labeling for preparing ground truth dataset if they are trained in a supervised way. Typically, the labeling of accelerometer and gyroscope signals are not easy because it is time series and usually have variable length, and in addition, it requires extra efforts to check corresponding video footage in order to validate activity labels.

We propose an unsupervised learning based deep learning method. The method does not require manual preparation of ground truth dataset. In our case, it becomes unnecessary to manually assign activity types to corresponding signal data of which quantity generally amounts to several tens of thousands. Our method consists of autoencoder and k-means clustering [16]. We train the autoencoder with time series signals for signal reconstruction, and extract only latent representations of inputs using the trained encoder. We then apply the clustering to those latent representations to determine a set of discriminative clusters. Hence, the activity recognition becomes the task of identifying which cluster the latent vector belongs to. It is like building an automatic classification pipeline without ground truth data.

The sensor data handling and analysis of our study is typically challenging because of the irregularity of the sensor attachment methods and areas. Firstly, the sensor attachment varies in location. We require the sensors be placed around any areas of chest and above clothes, without giving precise position and clothes condition. Secondly, the adhesion strength varies depending on the person who attaches it. Such loose condition is intentional because we worry that difficult restrictions on how to use the sensor make consumers hesitate to use it. Thus, our method should be able to consider variances coming from sensor placement.

The contributions of our work are as follows. From ten newborn and infant subjects, 69 MB of 3-axis accelerometer data and corresponding 4.3 GB of videos, which are 700 min long in total, were recorded. These types of activity data which are accompanied with real-time videos are rare. The other contribution is the unsupervised learning based model and the automatic activity labeling by the clustering. It helps avoid data labeling labor and automate the classification of confusing activities. We also propose a method of pre-processing data to facilitate normalization and reconfiguration of sensor signals by autoencoder. The preprocessed result has the characteristics of the original signal in a modified form.

The paper is organized as follows. We review similar HAR works in Section 2. We describe the collected data and propose the recognition method in Section 3, and discuss the performance evaluation in Section 4. Finally, we conclude our work and discuss future works in Section 5.

## 2. Related Works

HAR methods are largely divided into two main categories: video-based and sensor-based methods. Here, the sensor means the components which collects non-visual data. Thanks to rapid evolution of sensor technology, sensor pervasiveness because of IoT, and deep learning, HAR works are mostly sensor based. Privacy is another reason for the popularity of the sensor-based methods compared with the video-based methods

The sensors used to collect data related to newborn healthcare and wellness are very diverse [9,10]. They are different in the aspects of sensor type, placement, structure, and objectives. If sensors are divided by type, it can be classified into the following five: accelerometers, inertial measurement units (IMUs), magneto-inertial, pressure sensors, and flexible sensors. There are three main reasons for monitoring and analyzing newborn using sensors. The first reason is clinical needs. By using sensors to detect seizure-like behavior, physicians try to preemptively detect abnormal symptoms such as brain damage [17]. The second is to monitor the developmental stage. For example, by monitoring the strength of the hand grip, the growth stage of the newborn was estimated [18]. By monitoring the frequency and intensity of holding behavior between newborns and parents using a movement sensor, the effect of these behaviors on emotional development was analyzed [7]. The third is for safety purposes. To prevent sudden infant death syndrome, a sleep posture monitoring system was proposed [19]. Additionally, a wearable sensor detects a fall that can lead to head injuries [20] and there are things to alert drowning in the bathtub [4]. 

In addition to body movement, there have also been studies using various measurement objects. There are eye gaze tracking to monitor the newborn’s development [6], and to detect abnormal situations by analyzing CO_2_ in the breath [3]. This sensor-based monitoring system has the advantage that it can reduce the burden of dedicated monitoring personnel, and objective evaluation can be performed excluding subjective factors depending on the person [9,10]. Therefore, these sensor systems have been used not only in Neonatal Intensive Care Unit (NICU) of hospitals, but also at home as commercial products that can conveniently monitor newborn babies. These commercial devices have been used to monitor body temperature [17,18], monitor breathing while sleeping [16,19], and monitor heartbeat [20].

The adoption of wearable motion-sensing technology has increased for monitoring infant movements in hospitals and at home [8]. Due to miniaturized motion sensors and communication technology development, long-term monitoring of daily activity of infants with less disturbance becomes more practical than ever before. The traditional methods for monitoring infant health usually depend on the direct supervision of clinicians and parents, requiring dedicated labor resources. Even with such overhead, sometimes it is difficult for clinicians and parents to identify the infants’ potential physiological condition [21]. On the contrary, these wearable sensor systems are capable of conveniently and robustly monitoring infants in the NICU or at home.

The sensor-based HAR works are mostly built around deep learning based approaches as summarized in [1]. HAR is a kind of pattern recognition problem in essence. Thus, deep learning methods which have excellent ability in capturing complex patterns as features can offer effective ways for HAR. Before the adoption of the deep learning, human researchers were responsible for determining features which can distinguish different activities. With the deep learning, such efforts were delegated to powerful neural networks. In fact, the deep learning surpassed human ability by being able to extract complex but effective features which would have been impossible if humans did.

The deep learning provides various models suitable for HAR tasks. The models can be divided into five main types. These are fully connected network (FCN), convolutional neural network (CNN), autoencoder, Recurrent neural network (RNN), and hybrid models combining the four models. FCN was used from the early stage right after the introduction of the deep learning. However, humans were still responsible for determining features, while FCN performed classification based on the handcrafted features [22,23]. The FCN models sometimes failed for generalization because the human-determined features were too shallow to be general enough for various tasks.

CNN is one of the popular models for HAR because of its flexibility. The convolution, its representative operation, can be applied to not only for single dimension but also multiple dimension data. For example, 1D convolution can be applied for sensor readings of one-dimensional time series [24]. Even 2D convolution can be applied after converting one-dimension data to image-like two-dimensional form by dividing long one-dimensional data into equidistant segments and stacking them vertically [25]. For such conversion, domain knowledge is required to evaluate possible side effects from the conversion.

Autoencoder has the merit of unsupervised learning and having the chance to discover more general latent features than human-engineered ones. When the autoencoders are used, multiple instances of the autoencoders are stacked in a vertical way [26] to increase its feature expressiveness. RNN is strong in manipulating data which has temporal correlation. As RNN is slower in learning and requires larger computing resources than other models [27,28], it is rarely used alone but used as a sub-component in hybrid models. Sub-models in the hybrid models are responsible for its specialized tasks based on its strength. For example, the combination of CNN with RNN is the most popular [29,30], because they perform complementary roles; CNN is specialized in capturing spatial feature, while RNN is for temporal feature.

Anomaly detection, a sub-branch of HAR, also benefit from the deep learning. For example, if an autoencoder is trained with only normal data such as signals or images, it fails to reconstruct abnormal inputs. Thus, larger differences between input and output of the autoencoder can be used as indicators for anomaly. An autoencoder built with a set of LSTMs was proposed for anomaly detection in time-series data [31]. A similar approach [32] was proposed for detecting anomaly in robot operation by constructing variational autoencoder with LSTM. The most representative application of anomaly detection can be found in detecting abnormal ECG signals [33,34].

Non-deep learning approach is inevitable, if training data are insufficient and the labeling cost cannot beard. Besides such difficult situation, there are cases where algorithmic methods are efficient enough to show performance comparable to the deep learning. For example, a heuristic method for detecting discord in time-series data was proposed in [35]. Given an entire sequence, it selects a set of sub-sequences which are most different from a whole set of sub-sequences.

There were semi-unsupervised approaches [36,37,38] for activity recognition. Stacked autoencoders were trained in an unsupervised way to reduce noises and extract features from sensor data, but a supervised classification scheme was involved for activity recognition [36]. The training process of a convolutional autoencoder consists of two steps of which the first stage is unsupervised while the second is supervised [37]. The unsupervised training of an autoencoder was carried out for an auxiliary purpose such as noise sanitization in raw CSI data and high-level representative feature extraction [38].

The novelty of our proposed method is that its training proceeds in an end-to-end unsupervised way. Hence, not only the tiring work of labeling but also human-caused labeling errors can be avoided. In our case, the labeling work which checks a total of 700 min. sensor data and the corresponding video is very hard. Additionally, the labeling job becomes easily vulnerable to errors due to movement differences that are easy for humans to miss. Some errors can arise because of human factors; different people make different classifications on the data, and even the same person may classify differently depending on the time and one’s mood. Similar to other existing works, we use the autoencoder for the purpose of noise removal and high-level feature extraction of sensor data, but our work differs in that the whole process of training proceeds in an unsupervised way.

## 3. Data Gathering and Proposed Method

This section first explains the data collection process including video recording and the data format. Then, the proposed method for activity recognition using sensor data will be described.

### 3.1. Data Gathering

Data were collected while attaching a sensor device to subjects who are newborn and infants and, at the same time, taking a video. The sensor device was attached to the clothes without skin contact. The sensor device was certified as a medical device, but caregivers of the newborn babies preferred the contactless attachment. The device was placed to the upper chest area. The fabric between the sensor and the skin, the fabric over the sensor, etc. could not be forced to be identical to all subjects. The sensor device transmits data without buffering to the acquisition computer via Bluetooth communication.

**Figure 2:**
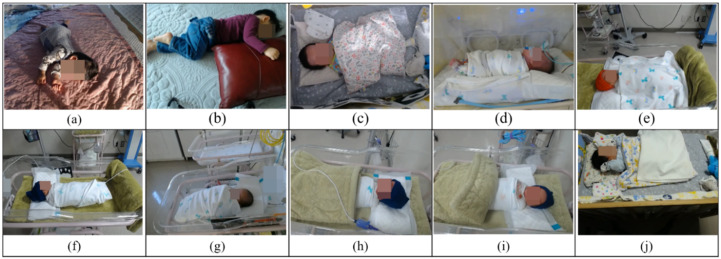
The captured samples of the videos taken while collecting the sensor data. To protect privacy, the body features of the subjects were blurred intentionally. Subject (**a**–**j**) correspond the (**a**–**j**) in Table 1.

While collecting sensor data, a video is taken at the same time. This is later used as a ground truth reference when determining whether the activity classification according to the sensor data is correct. The captured video as shown in Figure 2 is recorded with a webcam connected to the data acquisition computer at a speed of 30 fps in color with a resolution of 640 × 480, and it is possible to check whether the newborn or infants are moving by sight.

Table 1 shows the information about the subjects and the amount of the collected videos and the sensor data. Six out of ten subjects are newborn babies within 30 days of age. Additionally, except for one, they are infants under the age of one. In particular, (c) and (j) are the same subject but its data were collected at different times; thus, the actual number of different subjects is 9.

The video and sensor data are recorded as separate files, but the correspondence can be known by having the same time stamp on the file names. In addition, it is possible to synchronize the two data by managing the video frame index at the time the sensor data is received by the collecting computer. In order to efficiently store and manage collected data, and to minimize the risk of loss, separate files are created and recorded every 5 min.

**Figure 3:**
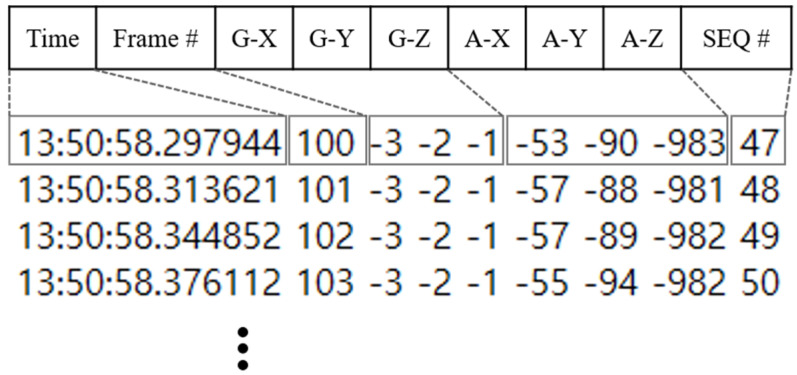
Examples of sensor data storage format and actual data.

The sensor data is saved as text in the format shown in Figure 3. One line corresponds to one piece of data received from the sensor device. The transmission frequency of the device is 40 Hz, but considering the delay in the data processing and storage process in the collecting computer, the actual reception frequency ranges from 36 to 40 Hz. The first field is a micro second precision time stamp at the time the data was saved. Frame # means the video frame index at the time point. Then, the six following numbers correspond to the gyroscope x, y, z axes and the accelerometer x, y, z axes, respectively. Only accelerometer values are used in the study. The last field, SEQ#, is an integer that circulates from 0 to 255, and is used as sequence data when the sensor transmits data, and is used for detecting data loss. Note that, to our surprise, when checking the continuity of the sequence number of the collected data in our experiments, no missing data occurred. This is presumed to be because the experimental environment was able to limit other wireless interfering devices to avoid data loss from interference. Nevertheless, robust and reliable imputation techniques are important enough to do independent research. We discuss them in future works.

**Figure 4:**
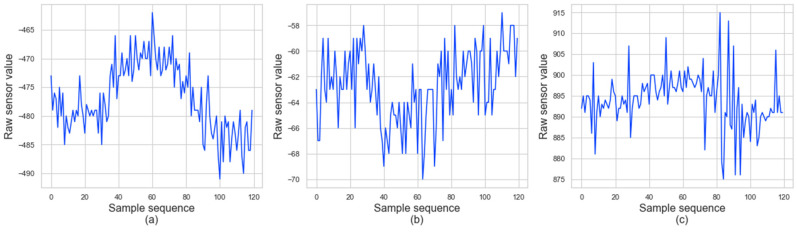
Example plots of collected accelerometer data for 30 s; the x, y, z axes correspond to (**a**–**c**), respectively. The x axis shows the sequence index of samples, and the y axis represents the raw acceleration value. This value is raw data from the ICM20600 chip, which was used to measure acceleration in the device. Gravitational force can be obtained by applying an additional calculation procedure to this value.

Accelerometer data can have values from −1000 to 1000 for each axis. The data represent the raw acceleration values from ICM-20600 chip [39], which contains 3-axis accelerometer and is embedded in the wearable device that we used for the experiments. Gravitational force can be obtained by applying an additional calculation procedure to this value. For example, Figure 4 shows the acceleration plot for each of the X, Y, and Z axes for 30 s. The difficulty in our data analysis is that the attachment position and direction of the sensor device may be different for each subject, and the device position and orientation may change over time due to movement. This difference is because the sensor attachment is affected by the newborn’s physical condition, other medical devices attached, and the skill level of nursing personnel. Therefore, we did not deliberately have restrictions on the attachment of the sensor device. This degree of freedom is difficult for data analysis, but has the advantage of being convenient for users.

### 3.2. Proposed Method Using Autoencoder and K-Means Clustering

**Figure 5:**
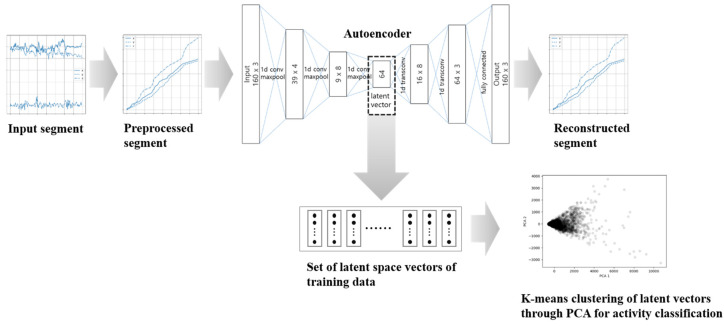
The overall architecture of the proposed model for activity classification.

The configuration and training process of the proposed model are shown in Figure 5. The model is divided into autoencoder and k-means clustering algorithm parts. In the training process, the autoencoder that aims to restore sensor data from the sensor data given as input is trained. When the training is complete, only the encoder part of the autoencoder is used to obtain latent space representation vectors of sensor data. These vectors can be viewed as low-dimensional features that contain the characteristics of sensor data. 

K-means clustering is used to automatically classify the latent vectors. When the clustering is applied, in order to determine the optimal number of clusters $n_{o}$, the intra-cluster variation is calculated while increasing the number of clusters from 2 to n, and $n_{o}$ is determined as the point where the variation rapidly decreases. In addition to the advantage that autoencoder can reduce the amount of calculation by dimension reduction, it is also helpful in improving classification performance by k-means clustering. For instance, in our experiment when clustering is applied to the encoded result, the silhouette score is increased by about 50% compared to the other case when applied to the preprocessed raw data, which will be discussed in the next section.

#### 3.2.1. Preprocessing

The input data to the model is a segment having 160 elements, each of which is a vector of length 3 and corresponds to x, y, z values of the acceleration sensor. Therefore, the dimension of the segment is 160 × 3 and its duration is about 4 s long. Given a whole sequence of accelerometer data, we create the segments by sliding window by 40 elements, which corresponds to one second long. Therefore, two consecutive segments overlap 120 elements, which corresponds to 3 s long and 75%.No principle exists regarding segment sizes and overlap amount. It is believed that decreasing the segment size enables faster activity detection in addition to reduced resources and energy needs, while large windows are considered for recognition of complex activities [40]. For instance, a public dataset of UCI for adult activity recognition [41], which is for classifying six types of behaviors such as walking, sitting, standing, etc., provides 2.56 s segments with 50% overlap between neighboring segments. Our segment size of 4 s is longer than that of the UCI because, rather than detecting fast movements in adults, our case is to work with slow movements in newborns. By increasing the overlap to 75%, we tried to reduce the occurrence of activity that could be missed at the segment border.

The segments go through a pre-processing process before it is fed into the autoencoder. The core effect of pre-processing is to change the sensor signal, which has the form of a wave shape moving vertically, into a monotonically increasing form. Its purpose is twofold; it reduces variances in sensor values due to different sensor position and direction, and transforms the data into a form that the autoencoder can reconstruct more easily. The first objective is similar to normalization commonly seen in deep learning. For this, we subtract the average of the elements from the segment in an element-wise way as shown in Equations (1) and (2). As a result, the input segment S is converted to Z.

(1) $$S=\;\left\{s_{0},s_{1},\ldots .,\;s_{n-1}\right\}\;\mathrm{where}\;s_{i}={\left[x_{i},\;y_{i},\;z_{i}\right]}^{T}$$

(2) $$Z\;=\left\{z_{i}\;|\;z_{i}=s_{i}-\mu \;|\;0\leq i<n\right\}\mathrm{where}\;\mu =\frac{\sum^{\;}s_{i}}{n},z_{i}={\left[x_{i}^{\prime },\;y_{i}^{\prime },\;z_{i}^{\prime }\right]}^{T}$$

The second objective is motivated by our observation that the autoencoder in our case can reconstruct monotonically-increasing sequence more easily than oscillating one. For instance, we found that, when using the input pre-processed in the increasing form, the reconstruction errors of the autoencoder was reduced by more than 70% compared to the case otherwise. Even if the shape of the signal changes due to preprocessing, in order to maintain the characteristics of the motion contained in it, we use absolute difference $d_{i}$′s between z_i′s as shown in Equation (3) and then calculates′s which is cumulative sum of $d_{i}$′s as in Equation (4). Note that both Equations (3) and (4) are applied in an element-wise way.

(3) $$D=\;\left\{\;d_{i}\left|d_{i}=|z_{i+1}-z_{i}\right|\;0\leq i\leq n-1,\;d_{n-1}=d_{n-2}\right\}$$

(4) $$C=\;\{c_{i}\;|\;c_{i}=\;\overset{i}{\underset{k=0}{\sum }}d_{k},\;0\leq i<n\;\}\;$$

The resulting segment C is given as an input to the autoencoder, and its training continues until the loss of mean squared error, which is in fact the difference between the reconstructed segment R and the input segment C, is not decreasing any more. Then, the autoencoder is used to extract the latent vectors of C, and the k-means clustering is applied to classify the vectors into groups, which completes the model training process. All the parameters mentioned in Equation (1) to (4) are listed in Table 2.

#### 3.2.2. Autoencoder

**Figure 6:**
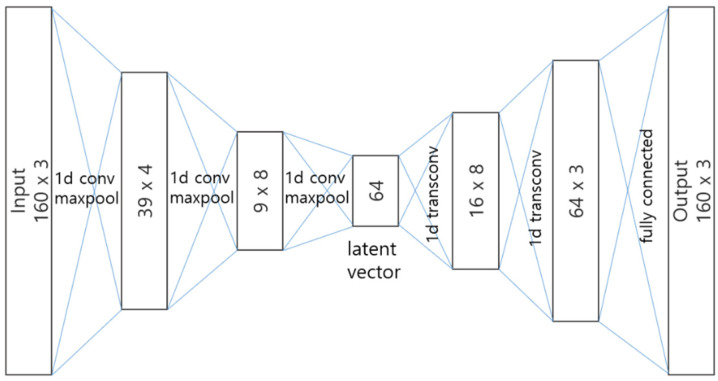
Autoencoder configuration which uses one-dimensional convolutions.

Figure 6 shows the configuration of the autoencoder and Table 3 shows the parameter sizes of the layers. Its purpose is to sanitize noise from input and extract high-level representation, just as conducted in existing methods. The encoder uses one-dimensional convolution considering that the segment is a time series. This has the effect of reducing the data dimension. On the contrary, the decoder recovers the original dimension same as the input by one-dimensional transposed convolution that plays the opposite role of the convolution. For an input segment having a structure of 160 × 3, the latent space representation vector becomes a vector of length 64. We also insert batch normalization, maxpool, and ReLU activation layers between convolutional related layers.

The architecture of the autoencoder is the result of trial and error and tuning. In the encoding stage, emphasis was placed on feature extraction, while the decoding stage put more emphasis on the recovery of the sequence. For example, in the input 160 × 3, 160 means the length of the sequence; thus, we wanted to have a decoding shape that proportionally increases the sequence length to 16, 64, and 160.

#### 3.2.3. Clustering

When using the k-means clustering, it is important to select an appropriate number of clusters. To determine this, we use the silhouette method [42]. The silhouette coefficient quantitatively determines how data is similar to its own cluster compared to other clusters. The score is between +1 and −1, and the larger the value, the more desirable. We calculate the silhouette coefficient of the k-means clustering for each different number of clusters , starting from $n_{c}=2\;$ and increasing by 1. When the coefficient value is high enough and the number of clusters is also large enough to represent the variety of activities, we select $n_{c}$ as the optimal number of clusters $n_{o}$.

**Figure 7:**
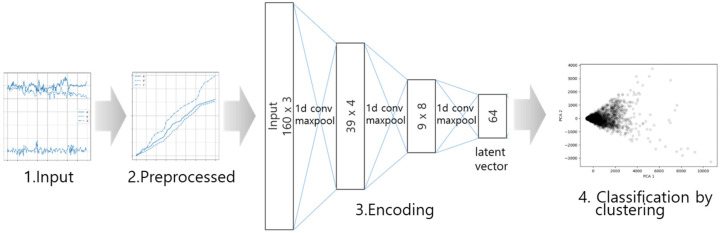
The classification procedure for activity classification.

Figure 7 shows the process of classifying activities using the trained model and the prepared k-means clustering. The encoder operation is performed on input data that have undergone cumulative pre-processing. The classification is performed by determining which cluster belongs to the resulting latent vector by using the k-means clustering.

## 4. Experiments for Performance Evaluation

For the model training, only the data of subject (d) of Table 1 were used because it contains all types of activities that we want to recognize. The data consist of 125 min of video and sensor signals. If we randomly select training data from all the subjects, there is a high probability that activities with low frequency, such as movement by external force, are not included in training data. Additionally, differences between newborns and infants should not be overlooked; it is obvious that the intensity of infant behavior will be greater than that of a newborn. However, in our experiment, the data of subject (b) were included in the experiment even if subject (b) was much older than other subjects. Since its data were acquired during sleep of subject (b), it seemed to be less problematic even if the difference between infants and newborns was considered. If the data were from waking movements, then the data should have been be excluded.

**Figure 8:**
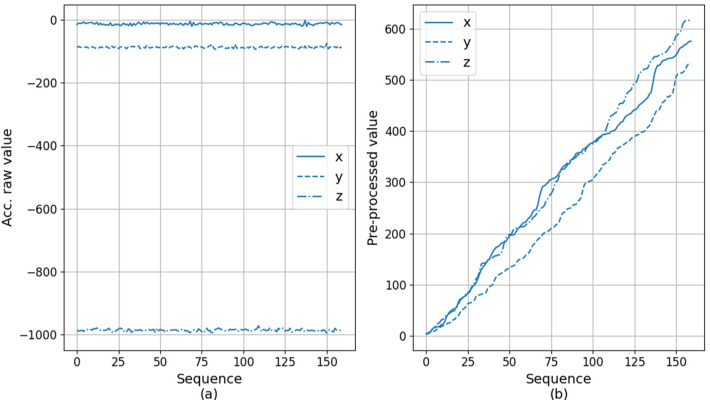
An example sensor data when a subject is in sleeping. Original acceleration data (**a**) and its corresponding preprocessed cumulative data (**b**).

**Figure 9:**
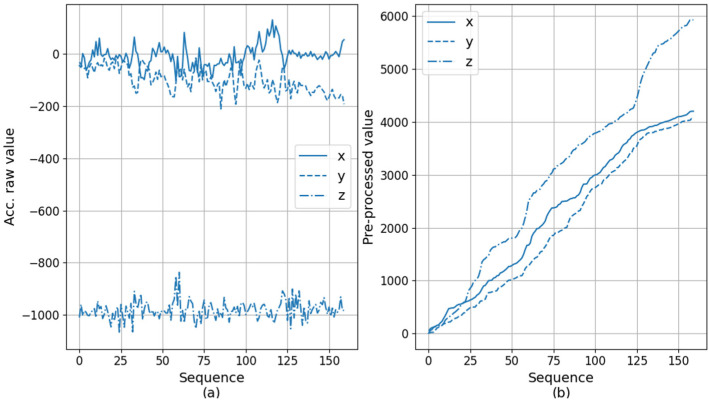
An example sensor data when a subject is moving. Original acceleration data (**a**) and its corresponding preprocessed cumulative data (**b**).

To prepare the training data, the segments of length 160 are created through slicing, and then the pre-processing is applied as described before. Figure 8 and Figure 9 show the examples of the original segment data and the corresponding pre-processed results. In the case of Figure 8, it is the partial data when the subject is sleeping. The original data plot in Figure 8a shows little change in all of x, y, and z axes. As a result, the plot of cumulative sum of Figure 8b increase gradually. The final cumulative sum reaches about 600. Figure 9 is when the subject moves largely while crying. Hence, it can be seen that the variation in all axes is large as shown in Figure 9a. Additionally, the steep rise of the cumulative plot is observed in Figure 9b, and the final cumulative sum reaches almost 6000 in the case of the *z*-axis, and both x and y reach around 4000.

**Figure 10:**
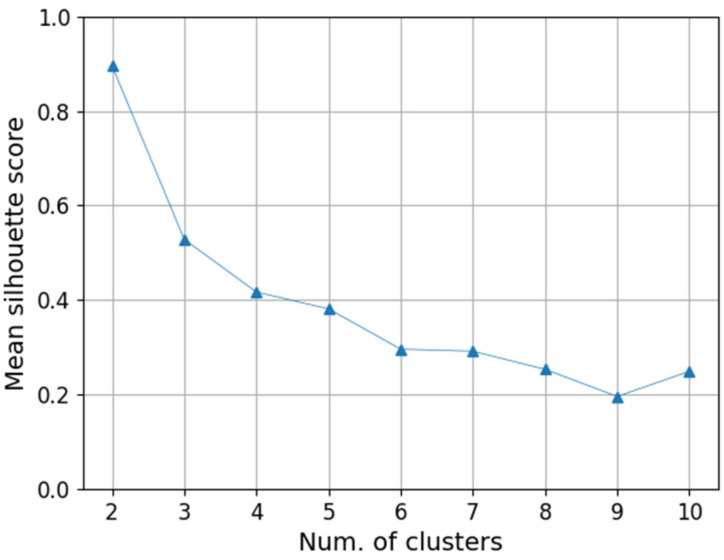
Silhouette scores according to the number of clusters. We use the scores to determine optimal number of clusters.

The autoencoder was trained using the prepared training data. As the loss for training, the mean squared error of the difference between the input and the autoencoder output was used. the training lasted 1000 epochs around which point the loss no longer declined. Using the trained encoder, the latent vectors for training data are extracted and classified by the k-means clustering. In order to determine the number of clusters which is directly related to the number of classifiable actions, not only the silhouette scores shown in Figure 10 but also the pre-screening observation of videos were considered. Browsing through the recorded videos, the newborn behavior was easily divided into three types: sleeping, awake, and being affected by external force. However, we wanted to add another classification in order to distinguish whether the awake behavior was simply awake in a comfortable state, or whether it was moving because of discomfort or pain. When examining the mean silhouette score, the threshold of 0.6 is the point where four behavioral classification was feasible. Since it was difficult to classify more than four behaviors even by watching videos, we did not consider the threshold lower than 0.6.

In deciding with the four actions was not only the result of observing the video, but also the necessity of this classification was explained in the referenced works. In order not to break the sleep rhythm of the newborn, it is necessary to detect the non-sleeping state and perform newborn care during that time [21]. As there are studies performing fall detection [20], it is important to detect external stimuli. In addition, as there are studies to monitor drowning in bathtubs [4], it is important to detect movements of newborns during pain and inform their caregivers. Based on the results of these existing studies, it was decided to distinguish between sleep and non-sleep. In the case of non-sleep, it was important to distinguish between external stimuli and self-moving. Even when moving on their own, it was important to distinguish whether they were simply waking up and moving during discomfort or pain.

The resulting activity classification and detailed description are summarized in Table 4. To see what behavior each category corresponds to, we check the corresponding video clip. According to the classification, behavior is largely divided into sleep and non-sleep. In the case of non-sleep, it is further subdivided into an active movement that moves by waking oneself and an external force movement that moves by the touch of caregivers. The active movement is further classified into strong and weak according to the intensity of action. Strong movement is a state in which the subject wakes up and writhes while crying, while the weak movement is a state in which the subject wakes up, but it shows light movements in comfy state. The external force is caused by caring actions such as lifting a baby during the care of a nurse or guardian.

To evaluate the performance of the model, the data of the remaining 9 subjects are used as test data. The latent vectors for the test segments are obtained from the encoder and then classified by the k-means clustering. The correctness of the behavior classification is evaluated by comparing with manually prepared ground truth data. To create a ground truth label for the segments of sensor data, we created a separate tool. It has the function of showing a video clip along with corresponding sensor data segment and allowing human to assign labels manually. To minimize individual labeling differences and errors, two different people independently performed labeling on the same data. Additionally, the results were compared to adjust different labels after consultation.

**Figure 11:**
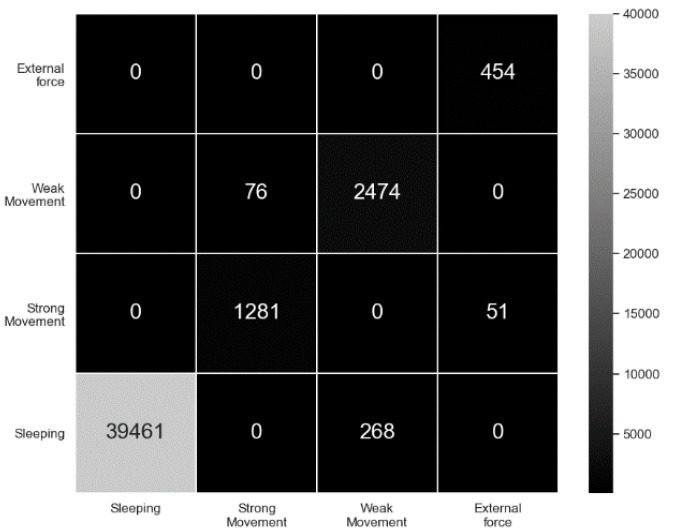
Confusion matrix for activity classification.

As a result, Figure 11 shows the confusion matrix for the classification evaluation. Additionally, Table 5 shows the precision, recall and F1-score for each activity. The recognition of sleeping behavior works bets with F1-score 0.99. The weak movement recognition shows the worst performance with F1-score 0.93 because it is easily confused with sleeping. External force also has low performance due to the confusion with strong movement. Overall, the mean F1-score for the four behavioral categories is 0.95, indicating that our model is robust enough for activity recognition. The balanced accuracy is 0.96, but its performance is partly due to the fact that the total number of sleeping segment is comparatively large and its recognition accuracy is high.

**Figure 12:**
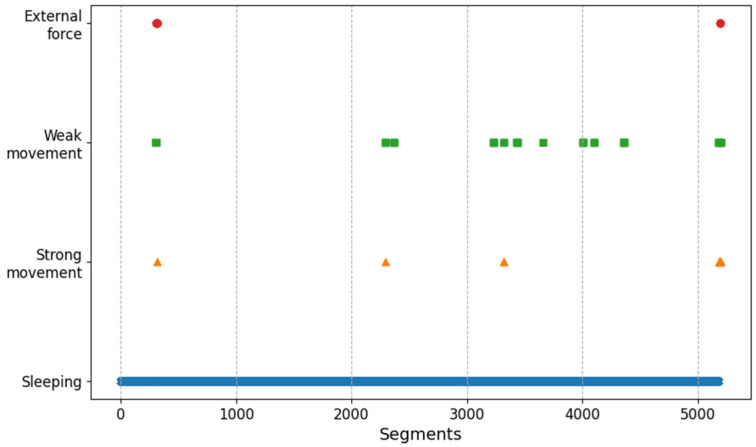
Activity plotting in time sequence of subject (a).

**Figure 13:**
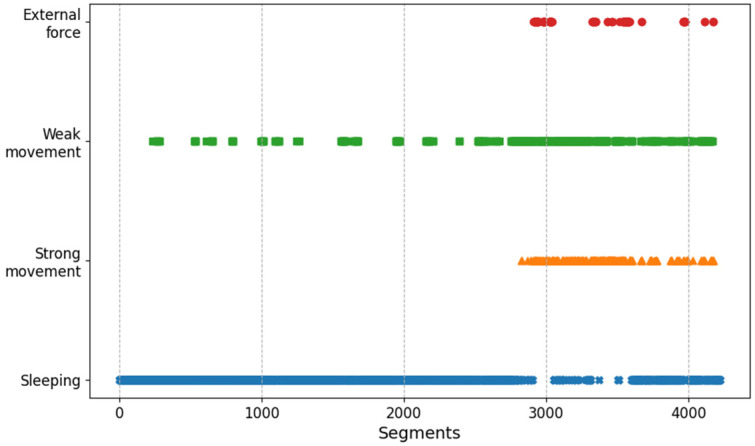
Activity plotting in time sequence of subject (d).

One of the applications of the activity recognition results is to indirectly evaluate a subject’s sleep quality. For example, Figure 12 and Figure 13 show the behavioral classification of subject (a) and (d) over time. Intuitively, it can be observed that the sleeping state of subject (a) is better than that of subject (d). In the case of subject (d), there exist various active movements and movements due to external forces after sequence 3000. Such analysis can help wellness care by providing information about subject’s sleep and non-sleep time. In addition, it can be used for risk prevention, for example, by sending an alarm to care givers when subjects show strong movement for a longer time than usual. 

## 5. Conclusions

In this study, we proposed an unsupervised end-to-end deep model for recognizing newborn behavior using acceleration values. We were able to automatically classify behaviors in the training process using autoencoder and k-means clustering. For this study, we collected 700 min of sensor data from 10 newborns and infants. Additionally, by taking video at the time of data collection, it was possible to secure ground truth reference for checking activity classification. Through this model, we found that the newborn’s behavior can be classified into four categories. Through the performance evaluation, the average F1-score was 0.96 and balanced accuracy was around 0.96.

In terms of computation, the size of the proposed autoencoder model is only 96 KB. Additionally, in K-means clustering, cluster determination itself does not have a large computational burden. It is meaningful that the proposed method is implemented on edge hardware. However, in general, we prefer to collect and analyze raw data in a cluster environment for the purpose of data accumulation and elaborate analysis. Therefore, considering the high performance of general clusters, the proposed method is sufficiently manageable.

We propose to use such activity recognition capability for evaluating the sleep quality, or monitoring the wellness and safety of newborn. In particular, monitoring body movement might be useful for valuing the sleep-wake rhythm of preterm infants. For instance, healthcare professionals are able to provide medical treatment or care while infants wake up, not interfering with their sleep pattern [43,44]. To this end, body movement can work as the key signal for professionals to start taking care of preterm infants and managing their environment. As for future work, since monitoring of the newborn’s condition is involved, robust and reliable imputation techniques are critical. There are various imputation techniques ranging from statistical techniques to using deep learning in recent years [45]. It is necessary to analyze the characteristics of various missing data patterns and verify the effectiveness of imputation techniques for them.

## Figures and Tables

**Table 1:** List of subject information and corresponding collected video and sensor data.

Sample	Gender	Age (Days)	Video		Sensor Data	Note
			Length (min.)	Size (MB)	Size (MB)	
(a)	F	360	20	341	2.05	
(b)	M	720	95	840	9.06	
(c)	M	180	10	75	1	
(d)	F	9	125	622	12.2	
(e)	F	2	95	601	9.81	
(f)	M	2	105	836	10.2	
(g)	M	16	135	923	13.6	
(h)	M	3	150	972	14.9	
(i)	M	4	85	459	8.62	
(j)	M	180	5	40	0.5	Same as (c)
Total			700	4453	69.83	

**Table 2:** The parameters used in Equations (1)–(4).

Parameter	Meaning
$s_{i}$	Original signal vector of accelerometer [x, y, z]
$z_{i}$	Mean-subtracted signal vector of [x, y, z]
$d_{i}$	Absolute difference between neighboring signal vectors of [x, y, z]
$c_{i}$	ACumulative sum vector

**Table 3:** The autoencoder architecture: layers and their parameter sizes.

No.	Layer	# of Param.	No.	Layer	# of Param.
1	Conv1d	28	7	ReLU/MaxPool1d	-
2	ReLU/MaxPool1d	-	8	ConvTranspose1d	520
3	Conv1d	72	9	ReLU	-
4	ReLU/MaxPool1d	-	10	ConvTranspose1d	99
5	Conv1d	272	11	ReLU	-
6	BatchNorm1d	32	12	Linear	10,400

**Table 4:** Activity classification.

Label	1st Classification	2nd Classification	Final Classification	Description
0	Sleeping			Subjects are sleeping without substantial movement.
1	Non-sleeping	Active Movement	Strong movement	Subjects are struggling with crying or in agony.
2			Weak movement	Subjects are awake and moving in comfy state.
3		External force movement		External force from nurses or care-giver is applied to subjects.

**Table 5:** Performance results of model classification.

Activity	Precision	Recall	Specificity	Balanced Accuracy	F1-Score
Sleeping	0.99	1	0.94	0.97	0.9966
String movement	0.96	0.94	0.99	0.97	0.9527
Weak movement	0.97	0.90	0.99	0.95	0.9349
External force	1	0.89	1	0.95	0.9468
Avg. (std.)	0.98 (0.018)	0.93 (0.049)	0.98 (0.027)	0.96 (0.011)	0.95 (0.026)

## References

[1] Wang J., Chen Y., Hao S., Peng X., Hu L. (2019). Deep learning for sensor-based activity recognition: A survey. Pattern Recog. Lett..

[2] Baker C.R., Armijo K., Belka S., Benhabib M., Bhargava V., Burkhart N., Der Minassians A., Dervisoglu G., Gutnik L., Haick M.B. (2007). Wireless sensor networks for home health care. Proceedings of the 21st International Conference on Advanced Information Networking and Applications Workshops (AINAW’07).

[3] Cao H., Hsu L., Ativanichayaphong T., Sin J., Chiao J. (2007). A non-invasive and remote infant monitoring system using CO 2 sensors. Proceedings of the SENSORS, 2007 IEEE.

[4] Nishida Y., Hiratsuka K., Mizoguchi H. (2007). Prototype of infant drowning prevention system at home with wireless accelerometer. Proceedings of the SENSORS, 2007 IEEE.

[5] Linti C., Horter H., Osterreicher P., Planck H. (2006). Sensory baby vest for the monitoring of infants. Proceedings of the International Workshop on Wearable and Implantable Body Sensor Networks (BSN’06).

[6] Schiavone G., Guglielmelli E., Keller F., Zollo L., Chersi F. (2010). A wearable ergonomic gaze-tracker for infants. Proceedings of the 2010 Annual International Conference of the IEEE Engineering in Medicine and Biology.

[7] Yao X., Plötz T., Johnson M., Barbaro K.D. (2019). Automated Detection of Infant Holding Using Wearable Sensing: Implications for Developmental Science and Intervention. Proc. ACM Interact. Mob. Wearable Ubiquitous Technol.

[8] Airaksinen M., Räsänen O., Ilén E., Häyrinen T., Kivi A., Marchi V., Gallen A., Blom S., Varhe A., Kaartinen N. (2020). Automatic posture and movement tracking of infants with wearable movement sensors. Sci. Rep..

[9] Zhu Z., Liu T., Li G., Li T., Inoue Y. (2015). Wearable sensor systems for infants. Sensors.

[10] Chen H., Xue M., Mei Z., Bambang Oetomo S., Chen W. (2016). A review of wearable sensor systems for monitoring body movements of neonates. Sensors.

[11] Infant, Newborn. https://www.who.int/infant-newborn/en/.

[12] Chois Technology Thermosafer. http://www.choistec.com/products/product_view.php?sq=142&code1=6&code2=.

[13] San-Segundo R., Zhang A., Cebulla A., Panev S., Tabor G., Stebbins K., Massa R.E., Whitford A., de la Torre F., Hodgins J. (2020). Parkinson’s Disease Tremor Detection in the Wild Using Wearable Accelerometers. Sensors.

[14] Thapa K., Al A., Md Z., Lamichhane B., Yang S. (2020). A Deep Machine Learning Method for Concurrent and Interleaved Human Activity Recognition. Sensors.

[15] Homayounfar M., Malekijoo A., Visuri A., Dobbins C., Peltonen E., Pinsky E., Teymourian K., Rawassizadeh R. (2020). Understanding Smartwatch Battery Utilization in the Wild. Sensors.

[16] MacQueen J. Some methods for classification and analysis of multivariate observations. Proceedings of the Fifth Berkeley Symposium on Mathematical Statistics and Probability.

[17] Chen H., Gu X., Mei Z., Xu K., Yan K., Lu C., Wang L., Shu F., Xu Q., Oetomo S.B. (2017). A wearable sensor system for neonatal seizure monitoring. Proceedings of the 2017 IEEE 14th International Conference on Wearable and Implantable Body Sensor Networks (BSN).

[18] Yamada T., Watanabe T. (2013). Development of a small pressure-sensor-driven round bar grip measurement system for infants. Proceedings of the the SICE Annual Conference 2013.

[19] Monbaby Sleep Monitors Official Website. https://monbabysleep.com/.

[20] Krenzel D., Warren S., Li K., Natarajan B., Singh G. (2012). Wireless slips and falls prediction system. Proceedings of the 2012 Annual International Conference of the IEEE Engineering in Medicine and Biology Society.

[21] Shimizu A., Ishii A., Okada S. (2017). Monitoring preterm infants’ body movement to improve developmental care for their health. Proceedings of the 2017 IEEE 6th Global Conference on Consumer Electronics (GCCE).

[22] Vepakomma P., De D., Das S.K., Bhansali S. (2015). A-Wristocracy: Deep learning on wrist-worn sensing for recognition of user complex activities. Proceedings of the 2015 IEEE 12th International Conference on Wearable and Implantable Body Sensor Networks (BSN).

[23] Walse K.H., Dharaskar R.V., Thakare V.M. (2016). Pca based optimal ann classifiers for human activity recognition using mobile sensors data. Proceedings of the First International Conference on Information and Communication Technology for Intelligent Systems.

[24] Chen Y., Xue Y. (2015). A deep learning approach to human activity recognition based on single accelerometer. Proceedings of the 2015 IEEE International Conference on Systems.

[25] Singh M.S., Pondenkandath V., Zhou B., Lukowicz P., Liwickit M. (2017). Transforming sensor data to the image domain for deep learning—An application to footstep detection. Proceedings of the 2017 International Joint Conference on Neural Networks (IJCNN).

[26] Almaslukh B., AlMuhtadi J., Artoli A. (2017). An effective deep autoencoder approach for online smartphone-based human activity recognition. Int. J. Comput. Sci. Netw. Secur..

[27] Edel M., Köppe E. (2016). Binarized-blstm-rnn based human activity recognition. Proceedings of the 2016 International Conference on Indoor Positioning and Indoor Navigation (IPIN), Alcala de Henares.

[28] Inoue M., Inoue S., Nishida T. (2018). Deep recurrent neural network for mobile human activity recognition with high throughput. Artif. Life Robot..

[29] Yao S., Hu S., Zhao Y., Zhang A., Abdelzaher T. Deepsense: A unified deep learning framework for time-series mobile sensing data processing. Proceedings of the 26th International Conference on World Wide Web.

[30] Ordóñez F.J., Roggen D. (2016). Deep convolutional and lstm recurrent neural networks for multimodal wearable activity recognition. Sensors.

[31] Srivastava N., Mansimov E., Salakhudinov R. Unsupervised learning of video representations using lstms. Proceedings of the International Conference on Machine Learning.

[32] Park D., Hoshi Y., Kemp C.C. (2018). A multimodal anomaly detector for robot-assisted feeding using an lstm-based variational autoencoder. IEEE Robot. Autom. Lett..

[33] Chauhan S., Vig L. (2015). Anomaly detection in ECG time signals via deep long short-term memory networks. Proceedings of the 2015 IEEE International Conference on Data Science and Advanced Analytics (DSAA).

[34] Wess M., Manoj P.S., Jantsch A. (2017). Neural network based ECG anomaly detection on FPGA and trade-off analysis. Proceedings of the 2017 IEEE International Symposium on Circuits and Systems (ISCAS).

[35] Keogh E., Lin J., Fu A. HOT SAX: Finding the most unusual time series subsequence: Algorithms and applications. Proceedings of the 5th IEEE International Conference on Data Mining.

[36] Gao X., Luo H., Wang Q., Zhao F., Ye L., Zhang Y. (2019). A human activity recognition algorithm based on stacking denoising autoencoder and lightGBM. Sensors.

[37] Seyfioğlu M.S., Özbayoğlu A.M., Gürbüz S.Z. (2018). Deep convolutional autoencoder for radar-based classification of similar aided and unaided human activities. IEEE Trans. Aerosp. Electron. Syst..

[38] Zou H., Zhou Y., Yang J., Jiang H., Xie L., Spanos C.J. (2018). Deepsense: Device-free human activity recognition via autoencoder long-term recurrent convolutional network. Proceedings of the 2018 IEEE International Conference on Communications (ICC).

[39] ICM-20600. https://invensense.tdk.com/wp-content/uploads/2015/12/DS-000184-ICM-20600-v1.0.pdf.

[40] Banos O., Galvez J., Damas M., Pomares H., Rojas I. (2014). Window size impact in human activity recognition. Sensors.

[41] Anguita D., Ghio A., Oneto L., Parra X., Reyes-Ortiz J.L. A public domain dataset for human activity recognition using smartphones. Proceedings of the 2013 European Symposium on Artificial Neural Networks (Esann).

[42] Rousseeuw P.J. (1987). Silhouettes: A graphical aid to the interpretation and validation of cluster analysis. J. Comput. Appl. Math..

[43] Calciolari G., Montirosso R. (2011). The sleep protection in the preterm infants. J. Matern. Fetal Neonatal Med..

[44] Colombo G., De Bon G. (2011). Strategies to protect sleep. J. Matern. Fetal Neonatal Med..

[45] Lin W., Tsai C. (2020). Missing value imputation: A review and analysis of the literature (2006–2017). Artif. Intell. Rev..

